# Genetic censusing identifies an unexpectedly sizeable population of an endangered large mammal in a fragmented forest landscape

**DOI:** 10.1186/s12898-015-0052-x

**Published:** 2015-08-25

**Authors:** Maureen S McCarthy, Jack D Lester, Eric J Howe, Mimi Arandjelovic, Craig B Stanford, Linda Vigilant

**Affiliations:** Department of Biological Sciences, Dana and David Dornsife College of Letters, Arts, and Sciences, University of Southern California, 3616 Trousdale Parkway, AHF 107, Los Angeles, CA 90089-0371 USA; Max Planck Institute for Evolutionary Anthropology, Deutscher Platz 6, 04103 Leipzig, Germany; Centre for Research into Ecological and Environmental Modelling, The Observatory, Buchanan Gardens, University of St Andrews, Fife, KY16 9LZ UK

**Keywords:** Habitat fragmentation, Genetic census, Ecological corridor, Chimpanzee, Population estimate, *Pan troglodytes*, Mark recapture

## Abstract

**Background:**

As habitat degradation and fragmentation continue to impact wildlife populations around the world, it is critical to understand the behavioral flexibility of species in these environments. In Uganda, the mostly unprotected forest fragment landscape between the Budongo and Bugoma Forests is a potential corridor for chimpanzees, yet little is known about the status of chimpanzee populations in these fragments.

**Results:**

From 2011 through 2013, we noninvasively collected 865 chimpanzee fecal samples across 633 km^2^ and successfully genotyped 662 (77%) at up to 14 microsatellite loci. These genotypes corresponded to 182 chimpanzees, with a mean of 3.5 captures per individual. We obtained population size estimates of 256 (95% confidence interval 246–321) and 319 (288–357) chimpanzees using capture-with-replacement and spatially explicit capture–recapture models, respectively. The spatial clustering of associated genotypes suggests the presence of at least nine communities containing a minimum of 8–33 individuals each. Putative community distributions defined by the locations of associated genotypes correspond well with the distribution of 14 Y-chromosome haplotypes.

**Conclusions:**

These census figures are more than three times greater than a previous estimate based on an extrapolation from small-scale nest count surveys that tend to underestimate population size. The distribution of genotype clusters and Y-chromosome haplotypes together indicate the presence of numerous male philopatric chimpanzee communities throughout the corridor habitat. Our findings demonstrate that, despite extensive habitat loss and fragmentation, chimpanzees remain widely distributed and exhibit distinct community home ranges. Our results further imply that elusive and rare species may adapt to degraded habitats more successfully than previously believed. Their long-term persistence is unlikely, however, if protection is not afforded to them and habitat loss continues unabated.

**Electronic supplementary material:**

The online version of this article (doi:10.1186/s12898-015-0052-x) contains supplementary material, which is available to authorized users.

## Background

Habitat loss and fragmentation are key threats to the survival of many species [1], with global deforestation resulting in the majority of remaining forest lying within 1 km of a forest edge [2]. Fragmentation can isolate populations, thereby reducing genetic diversity and population viability, which may result in local extinctions [3–5]. As wildlife populations face increasing anthropogenic threats, there is growing urgency to better understand how species respond to environmental disturbances. Although degraded habitats are often thought to have limited conservation value, many threatened species inhabit such environments [6]. Riparian forest fragments in particular can offer suitable habitat, providing dense resources to support wildlife [7, 8]. In addition, fragmented forests can sustain connectivity by linking larger populations, thereby enhancing gene flow and population viability [9–11]. Therefore, the potential of fragmented habitats to support viable populations must be carefully considered alongside the peril they pose to wildlife.

Large-bodied, wide-ranging mammals such as great apes are among the taxa most affected by growing habitat fragmentation. These species often live in unprotected areas, which are particularly vulnerable to forest loss and fragmentation [12, 13]. In East Africa, deforestation has led to increasing habitat fragmentation and poses a primary threat to the survival of eastern chimpanzees, *Pan troglodytesschweinfurthii* [14]. Eastern chimpanzees inhabit lowland and montane forest, woodland, savanna, and swamp forest habitats throughout various parts of East and Central Africa, with much of their current range occurring outside protected areas [14]. Three-quarters of chimpanzees in Tanzania are estimated to live outside national parks [15]. In Uganda, logging has led to a 37% reduction in forest cover between 1990 and 2010 [1, 16], and much of this deforestation occurred outside protected areas, leaving chimpanzees in such habitats vulnerable to local extinction [2, 17]. Similar patterns have also been reported for chimpanzees in West Africa [12, 18].

Because chimpanzees are an endangered species [19], it is essential to better understand their ability to persist in fragmented and degraded habitats. Moreover, precise estimates of the sizes and distributions of remaining populations are needed in order to establish research priorities and conservation management strategies. Such estimates can be challenging to obtain, however. Chimpanzee habituation allows for direct monitoring and hence precise censuses, but is a lengthy process which is necessarily restricted to small numbers of individuals, and may not be ethically appropriate or logistically feasible for many populations [20, 21]. Nest count surveys can be used to estimate the distribution and abundance of unhabituated chimpanzee populations. However, these survey methods may be inaccurate and lack the precision necessary to determine trends in population size [22–24]. Such studies are also arduous to carry out, as commonly used nest count methods rely on data regarding nest decay rates and nest building and re-use rates, which can be highly variable and are often unknown locally [22, 23, 25, 26]. Recently, camera trapping and passive acoustic monitoring have also been utilized to census apes [27–29]. However, these techniques are still in their infancy, while methods for efficiently automating individual identification are still in development [15, 30].

The challenges of accurately and precisely enumerating chimpanzee populations are similar to those posed by surveys of other rare and elusive mammal populations, including bears [31], gorillas [32–34], African elephants [35], Eurasian otters [36], and giant pandas [37]. These challenges have led to the widespread implementation of genetic censusing (e.g., in chimpanzees [38–40]), which relies on the characterization of individual DNA profiles derived from noninvasively collected samples [41]. The minimum number of individuals using the surveyed area is determined by the number of unique profiles, and resampling frequency can be used to estimate the number of animals that went undetected [42, 43].

Standard approaches for genetic censusing have relied upon accumulation curves and Bayesian estimators, along with more recent “capture with replacement” (*capwire*) models [43–45]. However, the population size estimates these methods provide cannot be converted to density estimates except by collecting ancillary data or making restrictive assumptions [46, 47]. Density is generally a valuable parameter because it can be compared across populations of varying size and geographic scope, and used as an indicator for behavioral ecology and conservation questions relating to, for example, resource density, group structure and dynamics, and hunting pressure [31, 48, 49]. Recently developed spatially explicit capture–recapture (SECR) models allow the density of geographically open populations to be estimated directly from spatially-referenced detections of individuals, by modeling probability of detection as a (usually decreasing) function of the distance between detectors or areas searched and individuals’ centers of activity [50–53]. SECR models are robust to spatial gaps in data collection [50, 52], which are common when sampling elusive species in degraded or mixed habitats.

In western Uganda, the approximately 1,200-km^2^ landscape of the Northern Albertine Rift separating the Budongo and Bugoma Forests illustrates such a degraded mosaic habitat. The government-owned Budongo and Bugoma Forest Reserves are each inhabited by over 600 chimpanzees, together composing approximately one-quarter of the estimated total chimpanzee population in Uganda (5,000 individuals [54]). The corridor between these forest blocks is a human-dominated landscape comprising mosaic riparian forest with villages, agricultural lands, and natural grasslands [55]. Most forests in this habitat are privately owned, but a few small government-owned forest reserves are present. The small forests in this region have been targeted for potential corridor enhancement given the vital role they may play for gene flow in numerous species throughout this region [56].

Despite the conservation potential of this habitat, few studies have examined the population size and distribution of its chimpanzees. A nationwide chimpanzee census used a nest count survey of forest fragments near the Bugoma Forest to extrapolate an estimate of ~70 chimpanzees in the corridor region [54]. Later, McLennan [55] found evidence of chimpanzees throughout the corridor habitat and estimated a total regional population of up to 260 individuals, an extrapolation derived from the estimated density of one chimpanzee community (Bulindi) in the corridor area [55]. Given the potentially vital role of this chimpanzee population in maintaining gene flow among chimpanzees of the Northern Albertine Rift, it is important to better understand the size and distribution of this population. The goal of this study was to use genetic censusing techniques to estimate the population size and distribution of this corridor population of chimpanzees in western Uganda. To do so, we estimated chimpanzee density using a spatially explicit model, as well as estimating abundance using both *capwire* and spatially explicit models. We further examined the number and spatial distribution of putative chimpanzee communities by analyzing the clustering of co-sampled genotypes. Additionally, because chimpanzees typically exhibit male philopatry and female dispersal, we examined the clustering of Y-chromosome haplotypes, which are paternally inherited and therefore can be used to reveal community affiliations [38, 40, 57].

## Methods

### Study area

**Figure 1:**
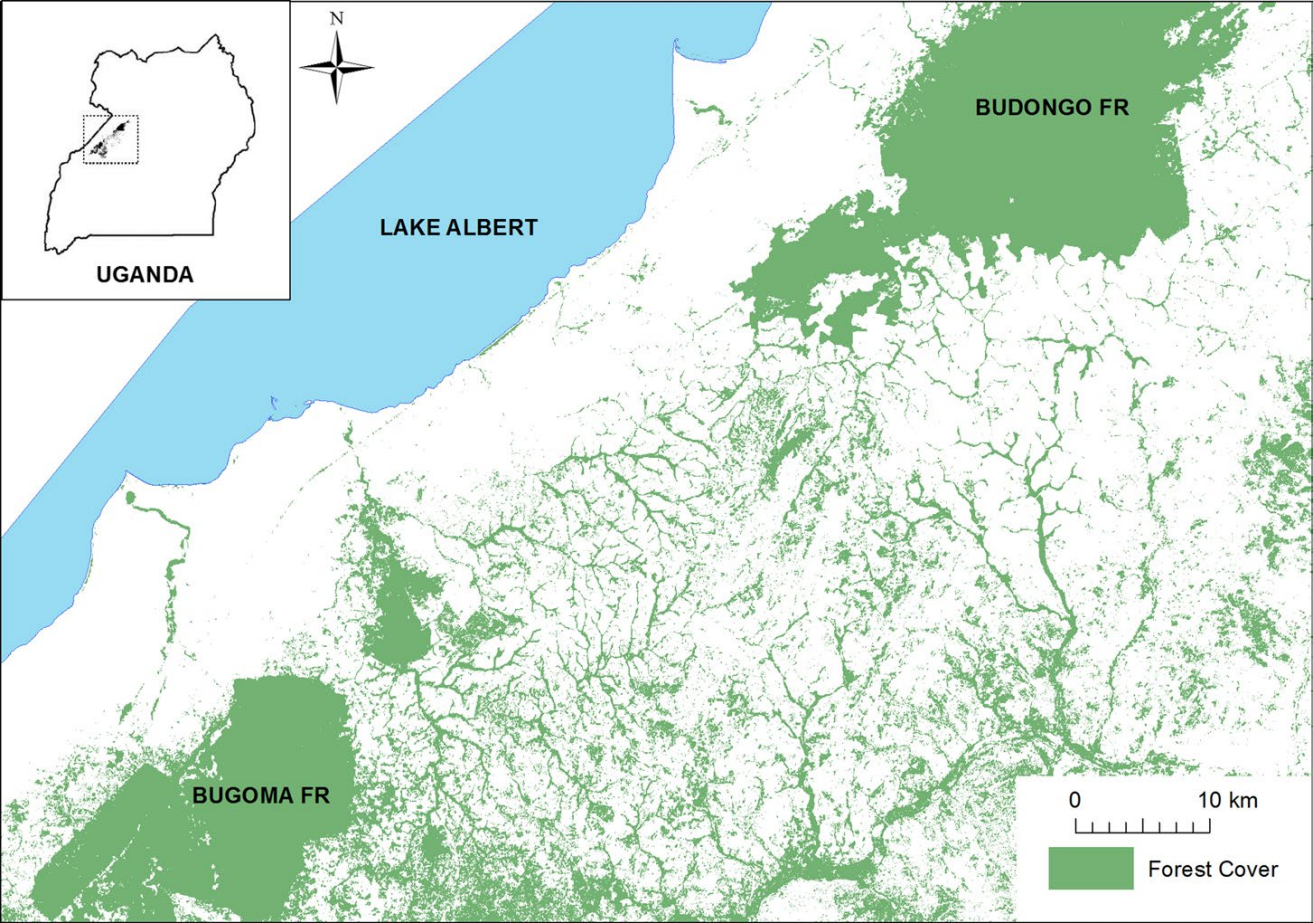
Map of the study area in Uganda. The inset map displays the landscape’s location within Uganda. *Green* indicates forest cover during the study period.

Data were collected in Hoima and Masindi Districts, Uganda, in the corridor region between the Budongo and Bugoma Forests (1°37′–1°68′N and 31°1′–31°6′E; Figure 1). Both forests are classified as medium-altitude, moist semi-deciduous forests [58, 59]. The Budongo Forest Reserve covers 428 km^2^, while the Bugoma Forest Reserve measures 411 km^2^ [54, 60]. The region between these forests, which broadly measures approximately 40 km long by 30 km wide, is a mosaic habitat composed of agricultural land, villages, riparian forest fragments, and grasslands. These riparian forests occur mainly along the Waki, Hoima, and Rwamatonga Rivers and their tributaries [55]. Pollen and climatic data indicate that the Budongo Forest has been a standalone forest block for thousands of years, and the region to its south likely existed as a natural mosaic habitat throughout that time [61]. In recent decades, however, human populations have grown substantially, leading to the extensive conversion of unprotected riparian forests for commercial and subsistence agriculture [16, 62].

### Genetic census methods

**Figure 2:**
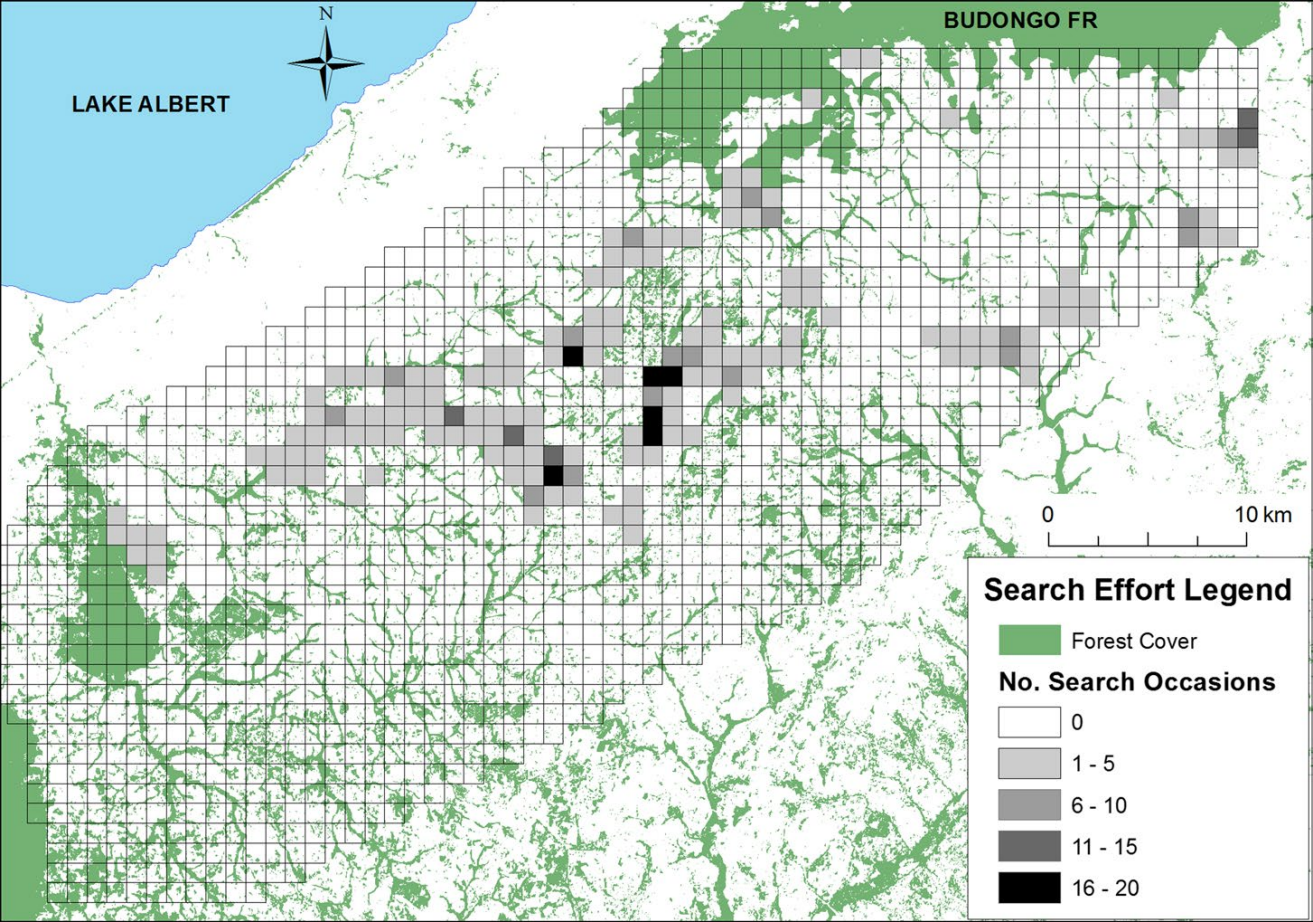
Map of search effort over the study area. One-km^2^ grid cells are overlaid over the corridor region between the Budongo and Bugoma Forests. *Gray* shading indicates relative search effort in each cell, with the number of search occasions (days) binned. Search effort was not available in the Bulindi area, where samples were collected during concurrent long-term research.

Chimpanzee fecal samples were collected noninvasively throughout the study area from October through December 2011 and October 2012 through September 2013. Samples were collected throughout the region, with a focus on searching riparian forest fragments for evidence of chimpanzees. Information on chimpanzee presence was also provided by McLennan [55] and by informal discussion with local inhabitants. It was not practical to employ strictly systematic survey methods in this human-dominated habitat comprising mainly privately owned farms and villages. Instead, search effort in forest fragments was centered around village boundaries, which typically encompass settlements, farmland, and privately owned forests. In accordance with local customs, prior to searching a forest fragment we first gained permission from the chairperson of the village in which the forest fragment was located, and from individuals who identified themselves as landowners of the forest fragment. We used satellite imagery to identify the forest fragments located within the boundaries of a given village, and visited accessible and permitted forest fragments within the boundaries of that village. We divided the study area into a grid of 1 km by 1 km cells and recorded when any part of each cell was searched (Figure 2).

Chimpanzee fecal samples were typically easy to identify because of (1) their locations under chimpanzee nests and along trails, (2) their characteristic size, shape, and odor, and (3) the absence of other sympatric large-bodied nonhuman primates. Although olive baboons (*Papio anubis*) produce dungs that can superficially resemble those of chimpanzees (pers. obs.), they have been eradicated from many parts of the study area. When we suspected that a fecal sample was produced by a baboon, a small portion of the dung was collected for genetic analysis, while the remainder was collected separately and washed later that day in a 1-mm mesh sieve. Fecal samples of baboons were easily distinguished from those of chimpanzees by their differing odor and dietary components when washed through a sieve [63]. Any sample suspected to originate from a baboon rather than a chimpanzee was thus discarded following washing (n = 5).

Target sample sizes were determined by roughly estimating the spatial area of a putative chimpanzee community home range, based on direct and indirect evidence of chimpanzee presence, then multiplying by the previously estimated density of chimpanzees in the Bulindi study community within the corridor region (0.66 chimpanzees per km^2^ [55]). This estimate was then tripled to determine a target number of samples to be collected within that area, since at least three times the number of samples as expected individuals has been recommended to achieve a narrow confidence interval for population size estimates using mark-recapture methods [33, 43, 44]. Because additional information on chimpanzee presence was gained over the course of the study period, target sample sizes were adjusted as necessary. To help achieve this sampling goal and to ensure adequate resampling across fission–fusion chimpanzee communities, we attempted to search forests a minimum of once every 3 months, except where local research permissions were granted only for a limited time period.

We collected samples under nests and opportunistically along chimpanzee trails and at feeding sites. For each sample collected, a GPS waypoint was recorded with a Garmin GPSMap^®^ 60CSx. We recorded samples with unique identification numbers corresponding to GPS waypoints, and with party association data when applicable. Samples were recorded as belonging to a party when two or more same-age samples were collected within 30 m of each other. Distances were determined using GPS data and, when necessary, a laser rangefinder to ensure accuracy. We avoided collecting two samples under the same nest or in close proximity on trails, due to the likelihood of collecting redundant samples from the same individual and the possibility that closely deposited samples may have cross-contaminated each other. Samples were collected and stored according to the two-step ethanol-silica method described in Nsubuga et al. [64].

Data collection was carried out with the permission of the Uganda National Council for Science and Technology, the Uganda Wildlife Authority, and the National Forestry Authority of Uganda. Additional permissions were granted by local landowners where applicable, as described above. Because fecal sample collection was entirely noninvasive and required no contact with the chimpanzees, ethical consent was not necessary for this project.

### DNA extraction and amplification

Samples were stored in the field for up to 6 months prior to arrival at Max Planck Institute for Evolutionary Anthropology, Leipzig, Germany, where they were then stored at 4°C prior to extraction. DNA was extracted using either the GeneMATRIX Stool DNA Purification Kit (Roboklon) according to manufacturer’s instructions or the QIAmp Stool kit (QIAGEN) with minor procedural adjustments [64].

We used autosomal microsatellite loci to determine individual chimpanzee genotypes. To do so, each DNA extract was first evaluated by simultaneously amplifying three autosomal microsatellite loci, along with an X-Y homologous segment of the amelogenin gene, used for sex determination [65], in a one-step multiplex polymerase chain reaction (PCR) (Table 1). For each reaction, we used 0.5 μL 2× Type-It Multiplex PCR Master Mix (QIAGEN) and 2 μL template DNA with the following optimized concentrations of each forward labeled and nested reverse primer [66, 67]: 0.03 mM amelogenin, 0.15 mM D18s536, 0.32 mM D12s66, and 0.30 mM D1s1622 in a total 10-μL reaction volume. Each PCR consisted of DNA extracts, as well one to two negative controls from each extraction, in four independent reactions. In addition, to monitor for consistency and possible contamination as is prudent when working with low concentration DNA derived from noninvasive samples, each PCR included one positive control from a chimpanzee extract with a known genotype and seven negative controls, which consisted of purified H_2_O instead of DNA. A PTC-225 Thermal Cycler (MJ Research) was used for PCR thermocycling as follows: denaturation for 5 min at 95°C; 45 cycles of 30 s at 95°C, 90 s at 58°C, and 30 s at 72°C; and a final extension for 30 min at 72°C, followed by incubation at 10°C. Each PCR product was then diluted 1:30 with purified H_2_O, and 27.4 µL of a 1:135 dilution of ROX labeled GENESCAN 400HD (Applied Biosystems) and H_2_O was added to size alleles relative to an internal standard. PCR products from all four loci were then electrophoresed using an ABI PRISM 3100 Genetic Analyser. We used GeneMapper version 3.7 (Applied Biosystems) to analyze the data.

**Table 1:** Autosomal and Y-chromosome microsatellite loci used in this study

Microsatellite locus	No. alleles	No. individuals typed
Autosomal		
D1s1622*	8	180
D12s66*	11	181
D18s536*	7	174
D1s1656	14	189
D2s1326	10	182
D3s2459	9	193
D3s3038	9	183
D4s1627	9	179
D5s1457	8	195
D5s1470	9	188
D7s817	9	188
D7s2204	8	179
D10s676	7	188
D11s2002	7	188
Y-chromosome		
DYs439	2	74
DYs469	3	76
DYs510	2	74
DYs517	2	76
DYs520	4	76
DYs588	2	76
DYs612	5	76
DYs630	3	76
(DYs392)	1	N/A
(DYs502)	1	N/A
(DYs533)	1	N/A
(DYs562)	1	N/A
(DYs632)	1	N/A

Asterisks indicate loci included in the single-step test multiplex. Y-chromosome loci indicated in parentheses were tested but were not variable and thus were not used further.

DNA extracts that reliably amplified at a minimum of 3 of the 4 loci in at least 3 independent amplifications were then genotyped in triplicate at an additional 11 autosomal microsatellite loci (Table 1). Extracts that failed to meet these criteria were not amplified further. The additional 11 loci were amplified in a two-step multiplex PCR procedure as described in detail in Arandjelovic et al. [66].

At each locus, heterozygous genotypes were confirmed by observation in at least two independent reactions [66, 68]. Homozygous genotypes were confirmed when observed in a minimum of three independent reactions. Individual loci that failed to meet these criteria were instead coded with asterisks and were excluded from analyses. To further ensure that apparent homozygotes were not the result of allelic dropout, we calculated allelic dropout rates by locus after recording all alleles and confirmed that a maximum of two replicates was required at any locus to confirm homozygosity with 99% certainty (Additional file 1) [68, 69]. Thus, we exceeded this threshold and ensured minimal allelic dropout by confirming homozygotes only when alleles were observed consistently in three reactions.

### Determination of Y-chromosome haplotypes

To determine Y-chromosome haplotypes, we first used a two-step multiplex PCR to assess the variability of 13 human-derived Y-chromosome microsatellite loci in a test set of 29 male individuals (Table 1) [57, 70]. Eight loci were polymorphic, with at least two alleles present. Thus, the remaining 47 males were typed at only these eight variable loci, which is similar to the number of variable Y-chromosome microsatellite loci found in various other studies of chimpanzees [38, 57, 71, 72], bonobos [73], western lowland gorillas [74, 75], and humans [76–78].

### Discriminating chimpanzee genotypes

Individual chimpanzee genotypes were distinguished using an identity analysis in CERVUS 3.0.7 software [79]. Using the allele frequencies of the study population, we determined the minimum number of loci necessary to achieve a P_IDsib_ < 0.001, which would allow us sufficient power to distinguish among genotypes and determine with statistical confidence that two matching genotypes from different samples originate from the same chimpanzee rather than from full siblings. Matching genotypes were assigned a consensus name and composite genotype data. Up to four mismatches were permitted to flag potential matches despite genotyping errors. Any mismatch was therefore either resolved as a true match with corrected errors or as a true mismatch comprising distinct genotypes. For rare instances in which genotypes matched with P_IDsib_ > 0.001, the less complete of the two genotypes was eliminated from further analysis.

### Assignment of putative communities and Y-chromosome haplotype distributions

Putative chimpanzee communities were defined according to the spatial clustering of co-sampled genotypes. In other words, genotypes found in association with other genotypes, e.g., as part of the same nest group, were assumed to belong to members of the same community. Further, additional lone samples from those individuals, such as samples found singly on chimpanzee trails, were inferred to lie within the home range of that individual’s community [38]. Using spatial data from these genotype clusters, we constructed 100% minimum convex polygons using the Minimum Convex Polygon Plugin for QGIS version 2.4.0 software [80] to represent the minimum home ranges of communities based on genotypes found in association. Additional genotypes found within these polygons were also assumed to originate from members of the same community, since extensive spatial overlap among territories is generally not expected [81–83]. Y-chromosome haplotype distributions were analyzed using a median joining network constructed in Network 4.6.1.3 Software (Fluxus Technology Ltd), and were mapped according to putative community distributions to determine whether spatial clustering of Y-chromosome haplotypes occurred in agreement with putative community distributions.

### Abundance estimation

We estimated total and community-specific population sizes using capture with replacement (*capwire*) models [44]. We used a likelihood ratio test to evaluate whether the “even capture” model (ECM), which assumes all individuals have an equal likelihood of capture, or the “two innate rates” model (TIRM), which allows for individual heterogeneity, provided a better fit to each data set. We expected capture probabilities to vary among individuals due to spatially and temporally variable search effort and possibly other factors, so we selected the TIRM when the P-value for the test was <0.10. Where the TIRM was selected we tested whether partitioning the data into three groups further improved the fit. The test statistic used was the ratio of multinomial log likelihoods for a two-class vs. a three-class multinomial distribution of the capture counts [84, 85], and was evaluated at an alpha level of 0.05. Confidence intervals were estimated by parametric bootstrap [44].

**Figure 3:**
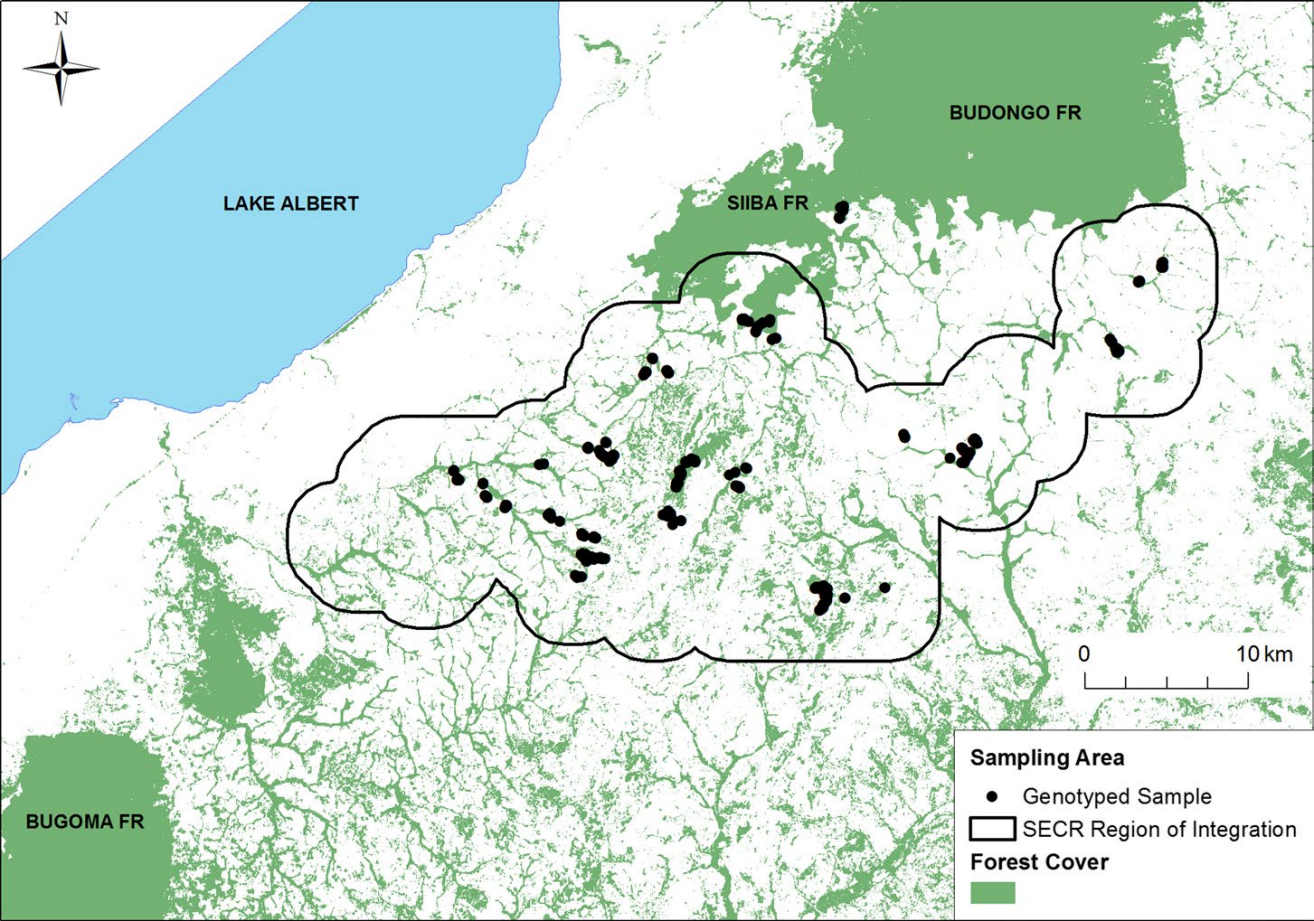
Genotyped sample collection locations across the study area. Not all samples are visible due to map scaling. The *black line* indicates the region of integration used in the SECR model. Samples outside the region of integration were collected in Siiba Forest Reserve and were excluded from analysis.

We also estimated chimpanzee density and population size using SECR models for area searches [52]. Search area polygons were defined as the perimeter of aggregations of adjacent, searched grid cells, or as individual cells if no adjacent cells were searched. We defined a contiguous region of integration as a 3-km buffer around these polygons, and verified that using a larger region did not affect estimates of model parameters. We defined two different integration meshes or “habitat masks” within this region in order to estimate densities both across the fragmented landscape and within the forest fragments. One mask treated the entire region of integration as suitable habitat where individuals’ activity centers could occur; for the other, we used spatial data describing forest cover [86] to exclude deforested areas from the mask. Multiple detections of the same individual were modeled as counts during a single sample [87]. Temporal variation in search effort was modeled as the average number of visits to the grid cells included in each search area polygon [88]. We assumed detectability declined with distance according to a half normal detection function, and that home range center locations were Poisson-distributed. We estimated detection parameters by maximizing the conditional likelihood for area searches, and density as a derived parameter from the fitted model [50, 52, 53]. We estimated population size by extrapolating the estimated density within forest fragments across forested habitat within the region of integration [89] (Figure 3).

All models assumed that (1) the population was demographically closed during sampling, (2) detections were independent events, and (3) individuals were correctly identified. *Capwire* models further assumed (4) geographic closure, and (5) that all individuals in the population of interest were at risk of detection. SECR estimates did not rely on assumptions 4 or 5 above, but assumed (6) that animals occupied approximately circular home ranges, the central location of which was fixed during sampling [51].

Analyses were performed in R version 3.1.2 [90] employing functions implemented in the “capwire” [84], “secr” [91], and dependent R packages.

## Results

### Genetic sampling and discriminating individual chimpanzees

We collected a total of 865 fecal samples over 633 km^2^ during the study period (Figure 3). Of these, 662 (76%) amplified reliably at a minimum of three of four test loci and were thus genotyped at an additional 11 loci. Based on our allele frequencies, we calculated that comparison at a minimum of nine loci was necessary to obtain a P_IDsib_ < 0.001 and thus confidently determine that identical genotypes originated from the same individual rather than two different individuals, including for example full siblings. Of the 662 genotypes, 459 matched exactly to one or more other genotypes and were merged to create consensus genotypes. An additional five genotypes were removed from analysis because they matched other genotypes with a P_IDsib_ > 0.001. The final genotype list consisted of 128 individuals identified in multiple samples (range 2–12) and 68 individuals genotyped once. For the analyses presented here, we removed 16 genotypes representing 14 individuals from a chimpanzee community in Siiba Forest Reserve, a continuous forest located to the south of the Budongo Forest (Figure 3). Since these genotypes originated from few samples in an under-searched area of continuous forest habitat, they were not informative or representative of the study population. The remaining genotypes represented 182 individuals, of which 111 (61%) were identified as female and 71 (39%) as male (Additional file 2). Consensus genotypes for these individuals were 95% complete, with 134 individuals typed at all 14 loci. Nine individuals were genotyped at fewer than nine loci, but their genotypes did not match any others and thus were retained in the data set. The mean number of captures per genotyped individual was 3.5.

### Putative chimpanzee communities and Y-chromosome haplotype distributions

**Figure 4:**
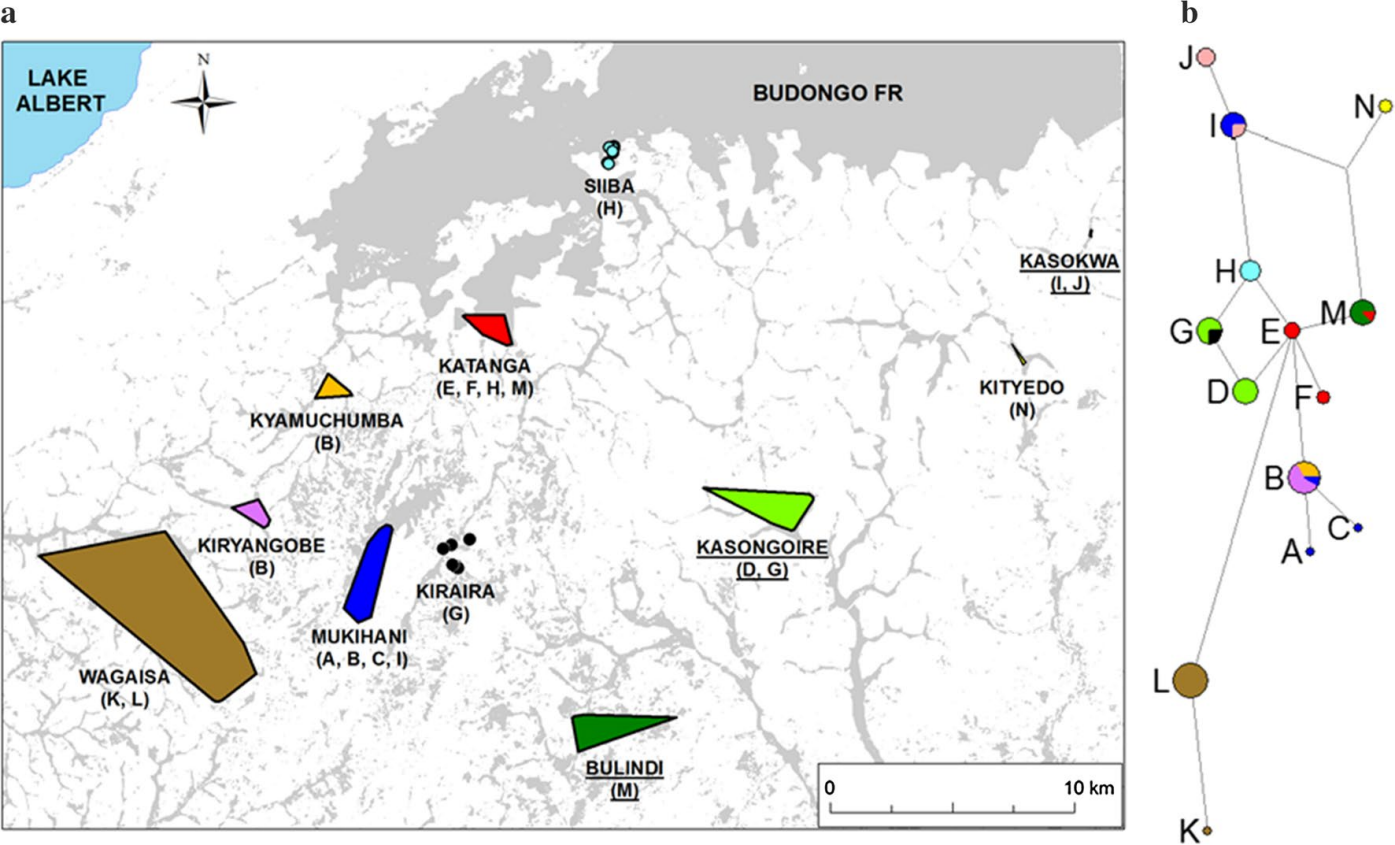
Putative chimpanzee communities (**a**) and associated Y-chromosome haplotypes (**b**). **a** Minimum convex polygons (MCPs) for genotyped samples found in association. Names of putative chimpanzee communities correspond to nearest villages and are listed below the MCP, with Y-chromosome haplotypes found in that putative community listed in *parentheses*. *Underlined* *names* indicate researched communities with preexisting data on approximate community sizes and home range extents. Each community is represented by a unique *color*. **b** Median joining network for the 14 Y-chromosome haplotypes. The relative similarity of haplotypes is represented by the lengths of branches, and the relative frequency of occurrence of each haplotype is indicated by the sizes of *circles*. *Colors* in haplotype *circles* correspond to putative communities in (**a**) exhibiting that haplotype.

By grouping genotypes from samples found together we found ten spatial clusters that were geographically distinct from one another, thus suggesting the presence of at least nine potential communities in the study area, along with one additional cluster, Kiraira. Community-specific population sizes estimated using *capwire* ranged from 5 to 48, and totaled 244 (Table 2). Data were insufficient to evaluate the fit of different models to data from Kiraira, and the upper confidence limit under the ECM was equal to the maximum population size we provided when fitting the model, indicating estimation problems. Figure 4 displays the distribution of putative communities.

**Table 2:** Community-specific *capwire* estimates

Group	n	N (groups)	95% CI	Monitoring estimate
Bulindi	17	19 (2)	17–21	19
Kasokwa	8	8 (1)	8–9	15
Kasongoire	28	38 (3)	31–56	34
Katanga	26	48 (1)	31–83	
Kiraira	5	5 (1)	5–200	
Kiryangobe	13	15 (2)	13–20	
Kityedo	16	18 (2)	16–21	
Kyamuchumba	11	13 (1)	11–19	
Mukihani	25	46 (2)	36–70	
Wagaisa	33	34 (2)	33–38	
Group-specific total	182	244		
Overall total	182	256 (3)	246–321	

Numbers of unique individuals genotyped (n) and population sizes (N) are shown with 95% confidence intervals (CI) for each putative chimpanzee community in the study area. The numbers of groups of chimpanzees with different probabilities of detection included in the estimate model appear in parentheses following the abundance estimate. Monitoring estimates refer to the number of chimpanzees reported during the study period for communities monitored for research or conservation (provided via pers. comm. as follows: Bulindi, Matthew McLennan; Kasokwa, Janette Wallis; Kasongoire, Geoffrey Muhanguzi). The sum of group-specific estimates, and the estimate of total population size obtained by pooling data from all communities for analysis, appear at the bottom.

From 76 total males (including those from Siiba) we found 14 Y-chromosome haplotypes, and these were 99% complete. Ten of these haplotypes were observed respectively only in single putative communities, thereby supporting community association data from genotype clusters. However, four haplotypes were shared among more than one putative community (Haplotypes B, G, I, and M; Figure 4). Overall, haplotypes shared a high degree of similarity as shown by their proximity in a median joining network (Figure 4).

### Abundance estimation

A likelihood ratio test supported the *capwire* TIRM model over the ECM model when fit to the full data set (ratio 132.4, *P* < 0.01). Partitioning into three groups was also supported (*P* < 0.01). We obtained a population size estimate of 256 (95% confidence interval (CI) 246–321]. The SECR estimate of average density across the fragmented landscape was 0.404 chimpanzees per km^2^ (SE = 0.033, 95% CI 0.34–0.47). The SECR density within forest fragments was 2.13 chimpanzees per km^2^, (SE = 0.17, 95% CI 1.8–2.5). The associated estimate of population size was 319 (SE = 17.6, 95% CI 288–357). The precision of the population size estimates, calculated as the CI width divided by the estimate, was 29% and 22% for the *capwire* and SECR estimates, respectively. The coefficient of variation (CV) of the SECR population estimate, measured as SE divided by the estimate, was 0.055.

## Discussion

### Abundance estimation

We employed two established estimators to determine the abundance of chimpanzees in a human-dominated landscape composed of small fragmented forests amid agricultural land. While a previous census estimated a population of ~70 chimpanzees in the study region [54], we obtained population size estimates of 256 and 319, more than tripling this previous estimate. These substantially higher estimates likely reflect the advantages of this approach over indirect abundance estimates, which can lack accuracy if little is known regarding habitat suitability and species distribution [22, 23]. Indeed, our estimates more closely resemble those of McLennan [55], who extrapolated chimpanzee density in the studied Bulindi community to similarly suitable habitat across the corridor region. One could alternatively explain the higher estimates as evidence of substantial population growth since the time of the previous census. However, given the slow interbirth interval of chimpanzees and the high rate of habitat loss throughout the region over the intervening years between surveys, this explanation seems highly improbable.

In addition to the improved accuracy of our estimates, our high recapture rate for chimpanzee genotypes across the study area also resulted in a relatively high degree of precision. Though adequate sampling is necessary to achieve precise estimates using mark-recapture methods [43, 44], this has proved challenging in numerous prior studies of great apes [38, 40, 92]. Our relatively high rate of resampling was aided by habitat heterogeneity, which led to a clustering of samples in confined areas of suitable forested habitat despite the large size of the total study area. We also directed our search efforts based on reports from local residents who live near the chimpanzees, which further benefited our sampling success rate.

Despite their relative precision, we found differences in the population size estimates provided by the *capwire* and SECR estimators, which may be an artefact of the differences in the specific quantities estimated by the models and their applications to a population with a heterogeneous distribution over a large spatial area. *Capwire* assumes all individuals were at risk of being detected. However, this may not have been the case, given the presence of spatial gaps in sampling and chimpanzees’ fission–fusion social structure, which could have caused us to resample parties of similar composition while failing to detect some community members, particularly where search effort was low. This may have caused underestimation of overall and group-specific population sizes when using *capwire*. To examine this possibility, we can assess the relative accuracy of our group-specific *capwire* estimates by comparing them with community size estimates based on observational data from communities being monitored for research or conservation. Of three such communities, two (Kasongoire and Bulindi) resulted in monitoring estimates that fall within the 95% confidence interval of our *capwire* estimates (Table 2). For the third community, Kasokwa, the TIRM estimate we obtained was substantially lower than the monitoring estimate. Spatial search effort in this region was relatively light, which may have resulted in identification of fewer genotypes from chimpanzees there and a corresponding underestimate as compared to Kasongoire, for which available search effort data reflect a broader spatial area search (Figure 2). Therefore, where search effort was greater and more broadly distributed, the TIRM estimate appears to be highly accurate, while in undersearched areas the TIRM estimate may fall short.

In contrast, by modeling detection probability as a function of distance between animals’ activity centers and areas searched, SECR models allow for the presence of additional individuals whose probability of detection is negligible because they spend most or all of their time outside the areas searched. However, this also means that the SECR model could have slightly overestimated population size if forest fragments far from the areas searched were, in fact, not occupied. We also note that the SECR region of integration included small portions of contiguous forest in the Katanga area (near Siiba Forest Reserve; Figure 3), such that our SECR model slightly overestimates the number of animals that rely exclusively on small forest fragments (between forest reserves). Despite the differences between estimators, the *capwire* and SECR estimates were qualitatively similar, with overlapping confidence intervals. Perhaps most importantly, the 182 distinct genotypes alone confirm a minimum corridor population size far exceeding that estimated in the previous nationwide census of chimpanzees in Uganda.

Additionally, the estimates presented here can be considered conservative if applied to the entire study area. The search area did not include some southern sections of the corridor, and we refrained from extrapolating density estimates to these areas since little is known regarding the current distribution of chimpanzees there (Figure 3). Chimpanzees have, however, been reported to inhabit forest fragments to the south and east of Wambabya Forest Reserve near the villages of Bugambe, Munteme, and Buhimba in Hoima District [54, 93, 94]. Additionally, Wambabya Forest has an estimated chimpanzee population of 136 individuals [54]. Our searches of the northern part of this forest yielded no evidence of chimpanzees, though relatively few searches could be allocated to this region. One additional chimpanzee community may also inhabit Rwensama Forest Reserve, just south of the Budongo Forest, but little is known regarding the size or range of this putative community. Future censuses in these areas may help clarify chimpanzee population size and distribution in Rwensama Forest Reserve, Wambabya Forest Reserve, and neighboring fragments of riparian forest.

Our estimates may also be conservative given that genetic censuses of great ape population size may tend to under-sample infants and juveniles due to the difficulty of finding their fecal samples. Based on a review of published demographic data from habituated chimpanzee communities, an average of 39% of a chimpanzee community is typically composed of infants and juveniles. If none of these individuals are sampled and are effectively at zero risk of detection, then the total size of a community or population will be underestimated. However, given our efforts to exhaustively search areas with evidence of chimpanzee presence, as well as our data indicating the small bolus size of some samples, we have reason to believe some infants and juveniles were sampled in our study population. If so, their detection risk would be elevated and our estimates should have adjusted accordingly to accommodate them.

Despite the advantages of these abundance estimators, potential model assumption violations should still be noted. Given the timescale of this study (15 months of sample collection over a total period of two total years), it is possible that we violated the assumption of demographic closure. However, given the slow life history traits of chimpanzees, whose average interbirth interval is more than 5 years [95], this is unlikely since relatively few deaths, births, or migrations into or outside of the corridor area would be expected to occur during this time. In addition, Arandjelovic et al. [33] found similar TIRM estimates when one longer-term (3 years) and two shorter-term (<1 year) sampling periods were compared for the same population of western lowland gorillas, suggesting the sampling period used in this study should not have strongly impacted abundance estimates. Community transfers would violate the assumption of fixed activity centers, but given the relative infrequency of female transfers in eastern chimpanzees [81, 96, 97], few instances are expected during the study period.

### Chimpanzee density in the corridor region

We used SECR models to estimate chimpanzee density both across the entire fragmented study area and within the forest fragments, obtaining estimates of 0.40 and 2.13 per km^2^, respectively. Estimated densities for chimpanzees in the Budongo and Bugoma Forests are approximately 1.3 and 2 chimpanzees per km^2^, respectively [54, 98]. Therefore, it appears that while the overall density of chimpanzees in the corridor region is relatively low, the density within forest habitat is much higher and may exceed that in continuous forest nearby. Chancellor et al. [39] found similarly high chimpanzee density (~2.1 individuals/km^2^) for eastern chimpanzees in a forest fragment of western Rwanda despite lower densities in montane rainforest nearby. Such findings may (1) indicate a crowding effect, whereby chimpanzee density is particularly high in small remaining areas of suitable habitat, (2) reflect the expected distribution of chimpanzees in a mosaic habitat with clumped resources, or (3) result from a combination of these factors. Previous estimates, however, have employed various non-genetic survey methods, thereby limiting our ability to draw conclusions by comparing densities across fragmented and continuous forests.

### Putative communities and Y-chromosome haplotypes

The spatial clustering of genotypes suggests the presence of at least nine different chimpanzee communities in the study area, in a non-overlapping distribution similar to that seen elsewhere among studied chimpanzees [81, 83]. Overall, Y-chromosome haplotypes show a structuring across putative communities, but 4 of 14 haplotypes are shared among more than one putative community. This overlap could indicate (1) remnants of older diversity from precursor groups in the region that eventually fissioned into different chimpanzee communities, (2) transfer events in which parous females with sons emigrated to new communities, thereby bringing with them new Y-chromosome haplotypes, (3) instances of extra-group copulations resulting in male offspring of different communities sharing the same Y-chromosome haplotype, or (4) mutations at microsatellite loci that caused closely related Y-chromosome haplotypes to converge into a single haplotype as defined using our markers. The reasons for its occurrence in this study cannot yet be determined but may result from one or a combination of these factors. A less plausible explanation is that shared Y-chromosome haplotypes indicate adult male dispersal. However, given that eastern chimpanzee males display a high degree of territoriality and intercommunity aggression [99, 100], this explanation seems unlikely, even in a degraded habitat. One additional possibility is that putative communities sharing a single haplotype are actually a single community. However, this explanation also seems unlikely given the high average recapture rate in this study, which often led to individuals being sampled among different party associations, as well as the large distances between some sampling clusters sharing a haplotype. For example, if we consider the maximum distance between sampling points for two males sharing the same haplotype (~34 km), and conservatively assume those points demarcate the outer edges of a single community home range, their circular home range would measure more than 900 km^2^ in size. The sharing of Y-chromosome haplotypes among multiple chimpanzee communities has also been seen elsewhere [71, 72, 101]. Future studies may better clarify the distribution of male philopatric chimpanzee communities across this region. Nonetheless, our results indicate likely conservatism in male philopatric territorial community structure despite substantial habitat degradation, a pattern that appears to hold for chimpanzees across numerous habitat types [102]. These findings support the behavioral data collected for chimpanzee communities in the region such as Bulindi, where fission–fusion community structure within defined territories appears intact despite widespread anthropogenic habitat destruction [55].

### Conservation implications

The results of this study suggest chimpanzees are both numerous and widespread in the human-dominated landscape between the Budongo and Bugoma Forests. This is perhaps surprising, given the paucity of forest habitat and the high human population density of 157 residents per km^2^ in this region [103]. However, chimpanzees in this area are known to utilize home ranges encompassing numerous forest fragments while feeding on a combination of natural and cultivated food resources [60, 104]. These forest fragments, which are largely riparian, are additionally known to harbor relatively high fruit tree density [8]. Indeed, riparian forest fragments in Central Africa have been noted for having high conservation value for chimpanzees and other species [7, 105]. In addition, chimpanzee survival under anthropogenic pressure is likely aided by their behavioral flexibility [106, 107]. Though their behavioral strategies in such habitats remain little understood, they include incorporating new (often human-cultivated) foods into their diets and adopting more aggressive or cryptic behaviors to mitigate human threats [21, 104, 108–110]. In western Uganda, their persistence is also attributable to relatively low hunting pressure, since Ugandans traditionally have not hunted chimpanzees for meat as in some other countries. However, customs are changing and chimpanzees are sometimes hunted for meat or killed as pests in Uganda, thereby making anthropogenic activities a threat to chimpanzee survival there [55, 111].

Despite anthropogenic pressures, these findings underscore the importance of greater investment in chimpanzee conservation in this region. A targeted solution such as translocating individual chimpanzee communities, as has been discussed [60, 112, 113], appears impractical given the large and broadly distributed population documented in our study. In contrast, our results suggest the potential may be high for a corridor enhancement project to benefit chimpanzees in this region [56], given that an increase in functional connectivity to the chimpanzee populations in the Budongo and Bugoma Forests would collectively impact 30% of Uganda’s total chimpanzee population (including the chimpanzee populations of both forests and the region between them). Any such project must be considered carefully, however. The need for firewood, building materials, and agricultural land are often cited as reasons for deforestation of the region’s unprotected forests [114]. Humans and chimpanzees also have a history of conflict interactions, given their close co-residence in this area [60, 112]. Stakeholder needs such as these must be taken into consideration to ensure the effectiveness of any conservation initiative. However, riparian forests play a key role in protecting rivers and the agricultural needs they support, so their conservation may increasingly be recognized as vital to the futures of both humans and other species locally. Additionally, habitat corridors may protect wildlife against the detrimental effects of climate change, thereby enhancing their value even further [115].

Our findings point to the value of conservation planning for unprotected areas with great potential to enhance gene flow and population viability among endangered wildlife populations. In this region as with many others like it, however, conservation action is urgently required. At least 450 km^2^ of forest is estimated to have been lost between the Budongo and Bugoma Forests from 2000 to 2010 [56]. Given the human population growth rate, this trend is likely only to change if concerted efforts are made to slow the rate of deforestation in the region. Though chimpanzees have proved surprisingly resilient to date in this habitat, their ability to withstand continued habitat losses, along with other threats to their survival, is highly uncertain.

## Conclusions

Using genetic censusing, we found a surprisingly large population of chimpanzees inhabiting largely unprotected forest fragments in western Uganda. The large size and widespread distribution of this population suggests it serves as a vital link between larger populations in the neighboring Budongo and Bugoma Forests. These results demonstrate the potential for forest fragments to serve as wildlife corridors, and for animal populations to be widely distributed in degraded habitats. Despite this potential, however, the habitat is rapidly being altered, and its capacity to support chimpanzees and other species may not persist unless the rate of habitat change is slowed considerably.

## Availability of supporting data

The data sets supporting the results of this article are included within the article and its additional files.

## Additional files

Additional file 1: Allelic dropout rates by locus. Allelic dropout rates for each of the 14 autosomal microsatellite loci used in this study. Dropout rates and the number of loci needed to achieve 99% certainty regarding genotypes were calculated as described in Arandjelovic et al. [66]. Additional file 2: Chimpanzee genotypes. Genotype data for each typed chimpanzee across the 14 autosomal microsatellite loci used in this study.

## Abbreviations

SECR: spatially explicit capture–recapture
PCR: polymerase chain reaction
ECM: even capture model
TIRM: two innate rates model
CI: confidence interval
CV: coefficient of variation
DAAD: Deutscher Akademischer Austausch Dienst (German Academic Exchange Service)
MCP: minimum convex polygon
